# Development and Implementation of a Public Health Event Management System, Nigeria, 2018–2024

**DOI:** 10.3201/eid3101.240379

**Published:** 2025-01

**Authors:** James Elston, Womi-Eteng Oboma Eteng, Chikwe Ihekweazu, Isabel Oliver, Everistus Aniaku, Anwar Abubakar, Christopher T. Lee, Emmanuel Benyeogor, Iain Roddick, Sophie Logan, Ebere Okereke, Leena Inamdar, Olusola Aruna, Rejoice Luka-Lawal, Christine Manthey, Lawrence Hinkle, Gloria Nunez, Emmanuel Agogo, Rabi Usman, Emmanuel Lucky Sunday, Muntari Hassan, John Oladejo, Ifedayo Adetifa

**Affiliations:** United Kingdom Health Security Agency, London, UK (J. Elston, I. Oliver, I. Roddick, S. Logan, L. Inamdar, O. Aruna); Africa Centres for Disease Control and Prevention, Addis Ababa, Ethiopia (W.-E.O. Eteng); Nigeria Centre for Disease Control and Prevention, Abuja, Nigeria (W.-E.O. Eteng, C. Ihekweazu, E. Ariaku, A. Abubakar, E. Benyeogor, R. Luka-Lawal, E.L. Sunday, M. Hassan, J. Oladejo, I. Adetifa); World Health Organization, Geneva, Switzerland (C. Ihekweazu); Resolve to Save Lives, New York, New York, USA (C.T. Lee, G. Nunez); Reaching the Last Mile Foundation, Abu Dhabi, United Arab Emirates (E. Okereke); Centers for Disease Control and Prevention, Atlanta, Georgia, USA (C. Manthey); CDC Foundation, Atlanta, Georgia, USA (L. Hinkle); Resolve to Save Lives, Abuja, Nigeria (E. Agogo, R. Usman)

**Keywords:** Event management systems, epidemiology, surveillance, One Health, Nigeria

## Abstract

Event management systems (EMS) are key tools for epidemic intelligence, integrating surveillance signals and incident response, although international standards to inform development are lacking. We describe the Nigeria Centre for Disease Control and Prevention (NCDC) SITAware, a software capable of operating with low internet bandwidth to generate notifications, reports, and spatiotemporal dashboards and provide event-level data for real-time accountability and postevent learning. SITAware was enabled by local institutional ownership, co-created at low cost, and integrated into existing workflows. In 2022, SITAware was used to manage ≈300 incidents, and NCDC implemented it subnationally. NCDC’s experience may inform EMS development and implementation in similar settings.

The COVID-19 pandemic and recent outbreaks of mpox, Ebola virus disease, and other diseases have highlighted the need to link public health data with action (*1*). During the past decade, many countries have made progress toward digitization of indicator-based surveillance (IBS) and event-based surveillance (EBS) systems (*2*,*3*). However, limitations remain, related to integration of signal and events data, multiplicity of systems, and interoperability.

Event management is the process of ensuring events of potential public health interest, as defined by the World Health Organization (WHO), are identified, risk assessed, documented, and reported early to inform rapid response actions to reduce event-associated illness and death (*4*). Effective event management requires the ability to follow and assess events and actions over the course of the event (*4*–*6*). Event management systems (EMS) can support event management by integrating data and workflows of surveillance and response actors. EMS can provide visibility in a single system for process escalation, including risk assessment and analysis, response initiation and coordination, as well as helping manage documentation of response and assigning and tracking of tasks. EMS supports public health institutions (PHIs) in meeting expectations concerning outbreak and incident oversight and response, as set out in the International Health Regulations (2005) (*7*,*8*).

WHO has developed an EMS as a centralized repository for event-related data to monitor and evaluate events and actions (*4*,*7*). Whereas guidance for event management and data frameworks for public health emergency operations centers (PHEOCs) have been published, standards and functionalities expected of country-based EMS are not clearly defined (*9*,*10*). Recent developments supporting epidemic intelligence are predominantly focused on the aggregation of source data for early detection of signals that could represent events, which includes the epidemic intelligence from open source tools (*11*,*12*). There is a lack of information in published literature regarding systems supporting subsequent steps in investigation, verification, and management of events. The inability to capture and share such information might compromise oversight, governance, accountability, and coordination of response and limits the opportunity for learning and continuous improvement (*5*). EMS can also help with integration of surveillance signals and event data from across sectors, such as animal, environmental, and human surveillance, in a One Health approach. EMS may also help PHIs improve, meet, and measure timeliness of detection, notification, and response, as proposed by the 7-1-7 target (*13*,*14*).

The Nigeria Centre for Disease Control and Prevention (NCDC) was established in 2011 with a mandate for preparedness, detection, and response to infectious disease outbreaks and public health emergencies and was codified in 2018 (*15*,*16*). Since inception, NCDC has strengthened infectious disease surveillance under the integrated disease surveillance and response (IDSR) strategy, improving laboratory diagnostic capacity and digitizing IBS and EBS systems (*17*). As a result, Nigeria has detected outbreaks of endemic and epidemic-prone diseases, including emerging or reemerging zoonotic pathogens such as mpox, Lassa fever, and yellow fever, and accelerated response to such events by strengthening oversight at national and state levels (*13*,*14*).

NCDC has invested in epidemic intelligence approaches and workflows to improve situational awareness and decision-making. NCDC, in collaboration with supporting partners including WHO, United Kingdom Health Security Agency (UKHSA), UK Department of Health and Social Care, United States Centers for Disease Control and Prevention (CDC), Resolve to Save Lives (RTSL), Bill and Melinda Gates Foundation (BMGF) and others, has continued to strengthen surveillance, IHR implementation, and EBS in Nigeria (*18*,*19*). In this article, we describe an EMS system, SITAware, that was developed collaboratively by NCDC and UKHSA to supplement the literature on EMS and inform future EMS development and implementation.

## NCDC Use Case and System Development

In 2018, we developed SITAware in response to a request by the chief executive officer of NCDC to support oversight and governance by improving visibility of public health events, their evolution, and actions and interventions in response. A need for real-time data sharing between states and NCDC during large-scale outbreaks and for an information repository to support institutional learning was expressed.

We tailored the SITAware system specification to address organizational priorities, user requirements for data entry, and specified technical requirements (Table 1). Under the terms of the interorganizational agreement, the system would be owned, hosted, and maintained by NCDC. The system needed to be stable, simple, and cost-effective to maintain and needed to function in the context of low internet bandwidth. Because the system would be reliant on staff of varied experience, it had to permit rapid data entry and demonstrate user benefit for sustained engagement. The system needed to create useful outputs for leadership, enabling information sharing with stakeholders, including state public health officials, ministries, departments, and agencies.

**Table 1:** Institutional and user system requirements for SITAware, the public health event management system developed and implemented in Nigeria, 2018–2024*

Perspective	Requirement	Considerations
Organizational	Low cost	Limited funding was available for development and the system had to be designed and implemented at a low cost relative to de novo software solutions. No additional funding for staffing or to address IT infrastructure was available initially
	Minimal hardware or software requirements	The requirement was to work within a given infrastructure. No funding was available for the provision of software or hardware
	Minimal need for maintenance	NCDC would host the system on its web server and maintain the system without additional resources
	Optimal performance over low internet bandwidth	In view of context, the system would need to perform well with low internet bandwidth
Users: data entry (e.g., surveillance staff)	Simple, easy-to-use interface	The system would need to be straightforward to use and easy to understand for users
	Minimal time commitment for the user	In view of competing demands for a limited workforce, the system should permit rapid data entry
	Benefit to user	The user should see benefit or reward in using the system relevant to their day-to-day role
Users: leadership	Overview of events, incidents outbreaks	An ‘at a glance’ overview or display of current incidents and outbreaks was required
	Auditable trail of events, evolution, public health response	A full history of entries to enable oversight and facilitate identification of lessons arising
Data Sharing	Real-time sharing of incident information within and across organizational boundaries	The system should permit real-time sharing internally between NCDC departments and teams and externally across organizational boundaries (e.g., between states and NCDC)

*IT, information and technology; NCDC, Nigeria Centre for Disease control and Prevention.

Rather than create a system de novo and being mindful of pressing needs and budgetary constraints (UKHSA allocated funding for development of ≈£30,000 during 2018–2019, with no funding for staffing or IT infrastructure), UKHSA undertook a review of relevant systems it operated that could be adapted as part of a bilateral surveillance strengthening program. The incident and outbreak logging (IoLog) surveillance system, used from 2009–2022 in eastern England, UK, for healthcare-associated outbreaks, was selected. IoLog is a web-based system with an SQL database housed on a UKHSA server that is accessed by a standard web browser. An unpublished evaluation of IoLog (during 2012–2015, comprising semistructured interviews and questionnaire survey of 41 users) indicated positive user experience, particularly relating to its simplicity and ease of use. UKHSA and NCDC worked in partnership in 2018 to develop the specification for the new adapted system that was developed by Camart Ltd (Cambridge, UK), leading to the launch of SITAware July 2018.

## Existing Surveillance Data Flows and Process Integration

NCDC oversees surveillance for human diseases by using IBS and EBS through the IDSR approach. IBS data systems have been digitized at the local government area (LGA) level and includes the use of the surveillance outbreak response management and analysis system, the Mobile Strengthening Epidemic Response Systems (an SMS-based tool for aggregated IDSR reporting), and District Health Information Software2, which provides immunization and health-programs surveillance (*16*,*17*,*19*). EBS systems include a call center and 2 web-based surveillance systems: Tataafo and the epidemic intelligence from open sources tool, used to aggregate digital media for signal detection (*17*,*20*).

**Figure 1:**
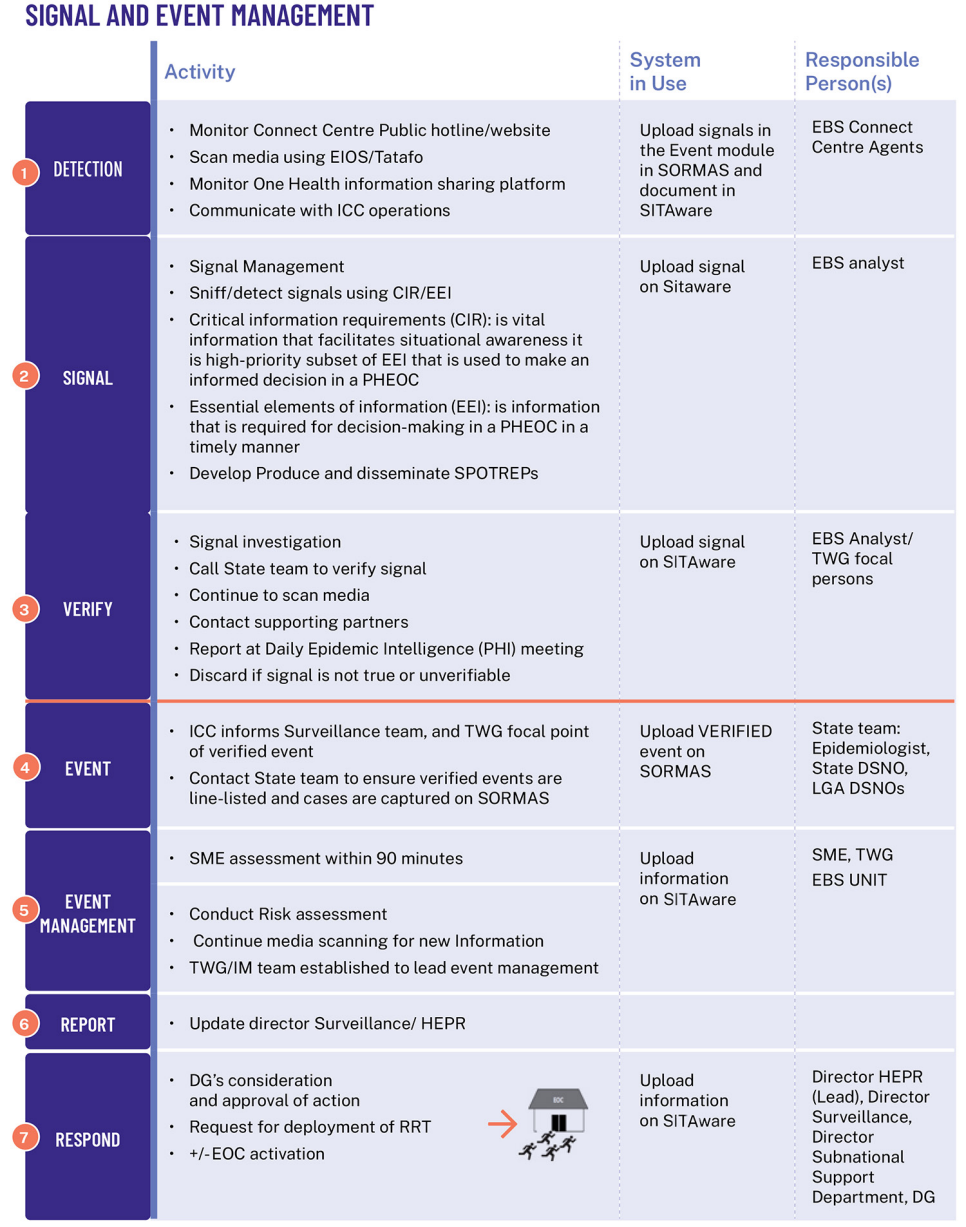
Nigeria Centre for Disease Control and Prevention signal management workflow. CIR, critical information requirements; DG, district government; DSNO, disease surveillance and notification officer; EBS, event-based surveillance; EEI, essential elements of information; EIOS, epidemic intelligence from open sources; EOC, emergency operations centers; HEPR, health emergency preparedness, response, and resilience; ICC, incident coordination center; IM, incident management; LGA, local government area; PHEOC, public health emergency operations centers; PHI, public health institutions; RRT, rapid response team; SME, subject matter expert; SORMAS, surveillance outbreak response management and analysis system; TWG, technical working group.

**Figure 2:**
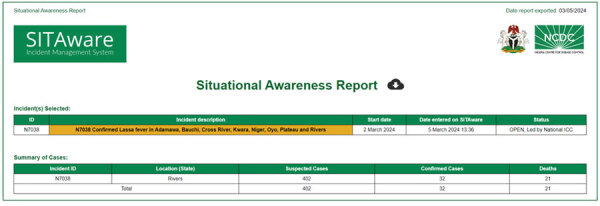
SITAware public health and event management system output example showing Situational Awareness Report header.

**Figure 3:**
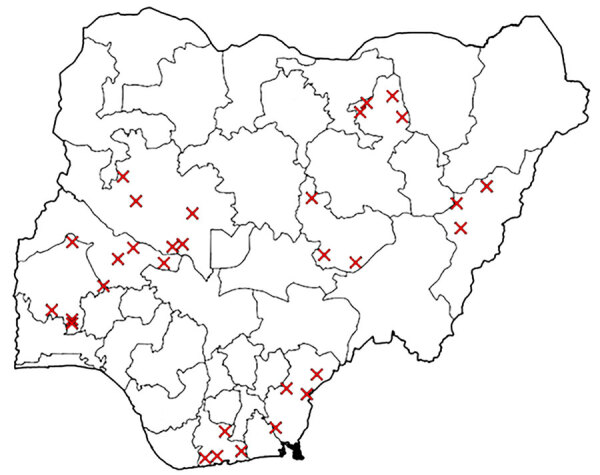
SITAware public health and event management system output example showing map within Situational Awareness Report indicating local government areas (districts) affected by an incident.

The use of SITAware has strengthened existing workflows and processes by providing effective coordination and risk assessment for rapid decision-making. For EBS, new signals originating from any system are entered immediately into SITAware and then uploaded to the surveillance outbreak response management and analysis system following state-level verification (Figure 1). EBS analysts apply predetermined criteria to determine the risk level of the signal and appropriate next steps or verification. Verified events are updated in SITAware and reviewed at daily public health intelligence meetings, which are visualized by using system outputs (Figures 2, 3) to enable joint appraisal and risk assessment in the NCDC incident coordination center (ICC).

**Figure 4:**
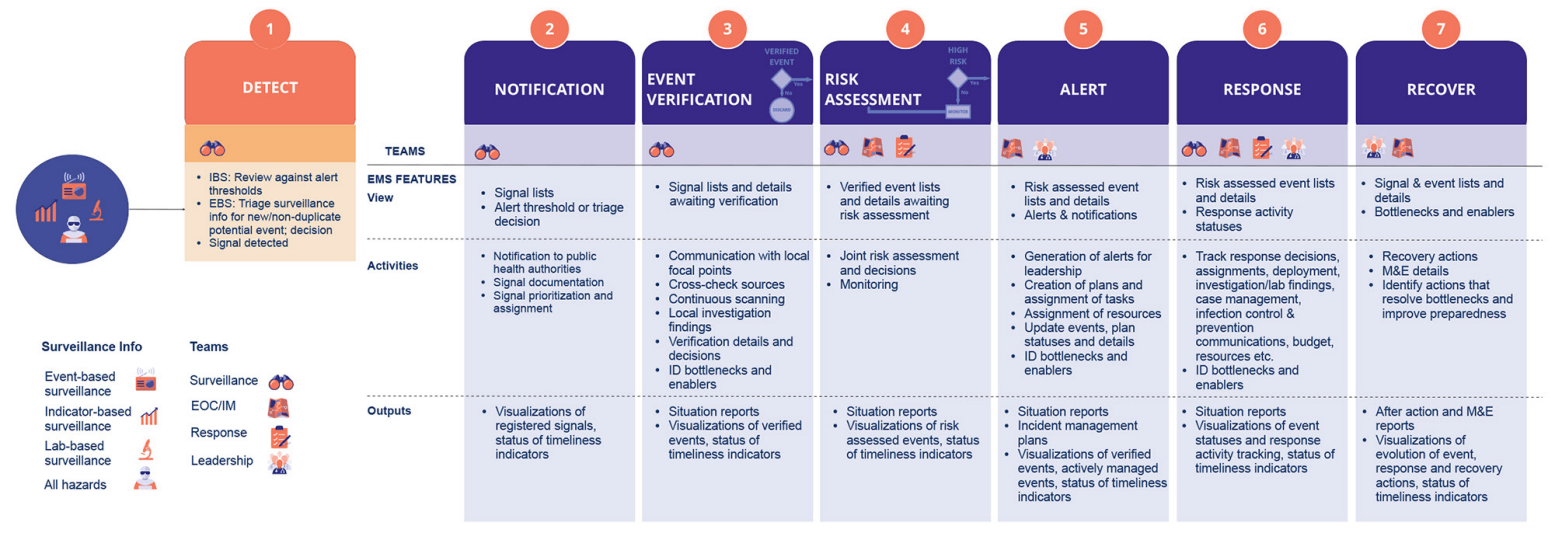
Nigeria Centre for Disease Control and Prevention event management system workflow. EBS, event-based surveillance; EMS, emergency management system; EOC, emergency operations centers; IBS, incident-based surveillance; ID, identify; IM, incident management; M&E, monitoring and evaluation.

The ICC serves as the PHEOC for coordinating response to major incidents at a national level, ensuring collaboration and partnership working with subnational PHEOCs and key stakeholders. NCDC has adopted the incident management system for managing events (*21*). SITAware plays a crucial role within this system by enhancing situational awareness, providing real-time data on incidents and a common operational picture for decision makers at various levels of the incident management system. ICC staff document risk assessment in SITAware within 90 minutes of a signal being entered onto the system to determine the nature and level of PHEOC response and then escalate reports as needed. Response actions and timelines are documented in SITAware. Outbreaks identified through IBS follow the same workflow without verification steps. A workflow and system integration summary are available (Figure 4).

## System Description

SITAware is a web-based EMS with specified user roles and configurations, designed to operate in low bandwidth environments. The system enables recording over an entire event, from detection to verification, risk assessment, response, and closure. Users record nature of the event (hazard type, pathogen, confirmation status, geography, and mode of transmission), timeliness metrics, case data, risk, and response measures (e.g., deployment by staff numbers and cadre, PHEOC activation status and level). SITAware enables users to share information between and within institutions. SITAware generates notifications and alerts by email, produces epidemic intelligence reports and dashboards, and provides a repository of events for accountability and post-event learning (e.g., after action reviews, 7-1-7 reviews) (*21*–*24*).

The SITAware infrastructure is hosted at NCDC on a Microsoft (https://www.microsoft.com) Windows server, IIS server, and SQL 2012 database server. The web application is written in ASP.NET C#, with some features, including the web-based dashboard, developed in JavaScript. Geographic data resolution is available at LGA level.

SITAware user configuration includes organization into domains that are team- and role-based; each domain has specific configurations for data sharing and edit control. State public health teams have state-specific domains. Domain administrators configure user access permissions, and individual users can view or hide domains to which they have access. Users are assigned to standard, administrator, or superuser categories. Administrators and superusers can create and manage user accounts. Superusers administer domains and manage and address user errors but are not permitted to view or edit incidents to preserve domain sharing integrity. A test-user category that does not interact with live domains is used for training.

SITAware was designed to have an intuitive user interface. Data fields are generated for data standardization using radio buttons, check boxes, list boxes, and free text fields. Entries may be updated any time; the user views a data entry screen prepopulated with the most recent entry. An archive of updates, edits, and user details is retained.

System outputs are available upon login. The user sees a list of open incidents ordered by time of entry that comprises details of disease, certainty, and location. An interactive dashboard map display indicates the nature, location, and relative size of incidents with filters permitting view by any given date range and relevant factors (e.g., deployment, PHEOC activation), linked to incident records. An interactive dashboard displays events, enabling the user to filter as required. A reports interface permits system search by using filters. Comma-separated value files can be generated as line lists of events (including disease, location confirmation, dates, case numbers and details, and PHEOC status). A printable output containing a complete record of entries can be generated for any event. A SitRep can be generated for single or multiple selected incidents (Figures 2, 3), summarizing details including nature, location, and case numbers, and provides an action log. Email notifications of new events or incidents can be generated by user-selected frequency.

## Rollout and Implementation

SITAware was launched after a 6-week pilot period and supported by system demonstrations, user trainings (surveillance and ICC staff), a system guide, and standard operating procedures. We tested the system utility by using functional exercises in 2019 and 2020. We completed integration of epidemic intelligence processes and workflows plus further optimization in 2019 incorporating feedback from partners. We further updated SITAware in 2020 to optimize outputs and use in daily health intelligence meetings. We implemented further user training events and mentorship to embed the system, build capacity, and ease use.

In 2021, NCDC commenced a subnational roll out of SITAware that included training and deployment to states. To date, public health teams in 35 of 36 states and the Federal Capital Territory in Nigeria have been trained and set up to use the system.

At the ICC and subnational PHEOCs, events logged on SITAware are reviewed daily for timely decision-making and response. The dashboard is typically projected, permitting real-time visualization of updates, key outbreak indicators, and response actions, providing a situational snapshot of major outbreaks. Use at subnational PHEOCs has strengthened incident response collaboration and coordination between the state and national levels.

During January–December 2022, SITAware logged 290 incidents across 32 states. Incidents related predominantly to infectious disease outbreaks, most frequently mpox (89 events in 24 states) and Lassa fever (84 events in 25 states). Incidents captured also included noninfectious disease events, including chemical hazards and unexplained deaths.

## Key Challenges in Implementation, Enablers and Lessons Learned

There was no budget for or initial allocation of dedicated staff roles. Training and implementation were supported by the UKHSA IHR program and existing NCDC staff. System maintenance required developer support.

User engagement with SITAware required substantial changes in working practice and culture, including greater emphasis on systematic recording with potential perception of increased oversight and scrutiny. The extent of change required was potentially underrecognized before implementation. Integration of SITAware into NCDC workflows required change management.

Because of the initial user engagement with SITAware, users were initially limited to a small number of engaged staff, and there was underrecording of events in the first year. Updating of events, including investigation, response actions, and event closure, was typically subject to delay.

The system remained stable, although local network and server issues periodically precluded access. In 2020, major data loss occurred because of a NCDC server malfunction in the process of migration to a secure external data center (where SITAware is now hosted). Some state users experienced problems relating to internet connectivity, requiring data reentry.

With support from partners, NCDC was able to allocate staff to provide dedicated support to SITAware including for rollout. An ASP.NET engineer was recruited to oversee maintenance, complete a technical review of the base code, architecture, and system security. An implementation officer was recruited to support training, user requests, and support use and optimization of outputs, which substantially improved institutional use of SITAware and led to more effective integration into daily epidemic intelligence workflows. Having a clear focal point for an EMS that ensures data are populated and outputs are generated for the right audiences could enable implementation in other countries.

Aside from the provision of staff, initial challenges rolling out the system could have been mitigated by more explicitly embedding SITAware into routine processes at the outset. Our experience highlights the importance of integration of new technologies and systems into existing workflows and the need to adopt a change management approach for users. Change management and embedding new practice requires individual buy-in, collective ownership, strong and devolved leadership, and development of new skills allied with continuous reinforcement and support; this approach was adopted.

We derived key lessons from the challenges experienced that included the need for explicit consideration of resourcing and allocation of roles to support implementation, engagement, and maintenance; adopting a change management approach; ensuring secure data storage and backup; and defined integration of systems into existing workflows. Despite those challenges, the system was implemented, embedded, and rolled out successfully. Key enablers for success (Table 2) identified by NCDC leadership, UKHSA, and partners included that the system was designed according to user and institutional needs; co-created harnessing user insights; owned by the national institution; adapted from an existing effective platform; and embedded within surveillance workflows and processes. Engagement was ultimately secured through providing technical and holistic user support. In addition, we consider that subnational rollout enabled national use, because there is more visibility and utility for national users with more events logged, so subnational rollout had benefits both at the state and federal level.

**Table 2:** Key enablers identified for the successful implementation of SITAware, the public health event management system developed and implemented in Nigeria, 2018–2024*

Key enabler for success	Description
Needs based and co-creation approach	SITAware was developed jointly by UKHSA and NCDC to meet NCDC’s identified needs. From initial conception to implementation of the system and system upgrades, the needs of NCDC and users were central to system design and technology adaption. Needs were clearly communicated and a process for periodic updates and progress review were set up.
Local institutional ownership of the system	Although SITAware was co-developed, NCDC ownership was ensured in contractual arrangements including full access to backend codes and other administrative privileges within the system. System installation and operation were embedded into existing institutional IT infrastructure using a locally defined protocol. Clearly defined ownership and leadership resulted in the implementation of the system and buy-in by users.
Leveraging an existing software	Adaptation of an existing tool ensured initial development was grounded in a demonstrable, concrete concept and that the system was useable from the outset adapting from a proven platform. Although adaptation may not be essential for the successful development of a new EMS, clarity on and emulation of functionality enabled implementation in this scenario.
Embedding within and enhancing surveillance workflows and processes	Ensuring the system would complement and enhance existing workflows and clearly defining the use case was important. In this scenario, SITAware was 1 of several concurrent enhancements to implement EMS though integration within existing processes and systems and was considered a critical enabler.
Providing of technical and holistic user support	Clearly defined leadership (including system champions and technical working group leads), provision of user training (initial user training and reinforcement), mentorship, supporting materials and technical support remain crucial to embedding and improving the use of SITAware and remains works in progress. Ensuring such provision at the outset and over the long term, ideally including consideration of additional and dedicated human resources to support implementation, is perceived as a critical success factor.

*EMS, event management system; IT, information and technology; NCDC, Nigeria Centre for Disease Control and Prevention; UKHSA, United Kingdom health security agency

## System Utility and Next Steps

A formal system evaluation of SITAware is pending, and we acknowledge those results would help to demonstrate usefulness and effect. Nonetheless, there are several initiatives where SITAware has been assessed directly or indirectly. A survey (Nigeria Centre for Disease Control and Prevention, unpub. data) conducted by NCDC in August 2024 evaluated the management and performance of the SITAware platform among PHEOC staff across 35 trained states and Federal Capital Territory in Nigeria. The survey aimed to assess the effectiveness of the platform in signal reporting, identify challenges in technology adoption, and measure the effect of internet accessibility on the platform’s usage. Key findings from this survey included a correlation between knowledge and use of the system and internet coverage: states with good internet coverage were frequent users demonstrating good knowledge of SITAware; states with poor internet coverage used the system less with lower awareness. The survey reported that the performance of the SITAware platform was generally considered excellent, and conclusions stated, “As of August 2024, the SITAware platform facilitated the reporting of 861 signals across various states, highlighting its critical role in supporting public health emergency operations” (Nigeria Centre for Disease Control and Prevention, unpub. data). The report emphasized the importance of addressing knowledge gaps through training and internet infrastructure.

The NCDC formal document use case, created in June 2024, highlighted that the SITAware platform was a source for public health intelligence and is used at the institutional level for decision making in emergency preparedness and response (Nigeria Centre for Disease Control and Prevention, unpub. data). The document also stated that the platform had robust stakeholder engagement and was a source of outbreak evaluation (Nigeria Centre for Disease Control and Prevention, unpub. data).

Other opportunities for SITAware assessment and evaluation have included training, formal simulation exercises, and after-action reviews. Ease of use and rapid, effective user engagement have been highlighted by those opportunities.

During the early stages of the COVID-19 pandemic, SITAware was valuable to document pandemic evolution in Nigeria, informing the pandemic response. Daily summary reports generated by the system were used to support situational awareness and decision making. SITAware enabled review of the national response to the pandemic and related major policy changes, including a COVID-19 Mid-Action Review (*25*–*27*).

NCDC leadership believes SITAware has met the needs and requirements specified at inception. Embedding the system into day-to-day activity, albeit with the need to overcome associated challenges, has effectively enabled event identification and management and enhanced partnership and collaboration with states. SITAware has addressed gaps at the intersection of public health and emergency management and secured coordination to inform resource allocation and response through provision of analytics, geographic information, and resource tracking.

NCDC is currently undertaking a technological review of SITAware and addressing requests from users, which could include adding further functionality (e.g., structured response data elements, project management for response tracking, use by animal and environmental sectors). 

## SITAware as a Prototype for National Event Management Systems

The experience in Nigeria has highlighted the need for national and subnational EMS to support epidemic intelligence workflows, provide data outputs, and ensure that decision-making is prompt and responses are accountable. On the basis of our experience, we believe SITAware can serve as a prototype for an EMS design that can be adopted by other countries, representing a workflow for signal and event management incorporating IBS, EBS, and One Health approaches (Figure 4). A wider rollout of SITAware is a potential option but would require updates for context and appropriate upgrades.

A national-level EMS should integrate outputs of existing surveillance systems to have representation and attribution at the event level. EMS would ideally be interoperable with (or combined in the same system with) core indicator or case-based electronics surveillance systems to update case data and automatically generate events when alert thresholds are passed or signals are verified. EMS would also serve as a data repository drawing from surveillance systems that may not be digitized, enabling user entry of events in other sectors or where full interoperability is not achievable. EMS should enable workflow integration and process support from detection to response, documenting risk assessments as events evolve. It would support timeliness milestone collection for outbreak emergence, detection, notification, and response, as proposed by the 7-1-7 target, which will support event management and real-time performance improvement approach for PHEOCs and national PHI. The system could support structured data fields for response actions, which in turn provide project management support that ensures visibility and accountability for responses. EMS should integrate with and complement emergency management systems and help coordinate response to major events.

## Discussion

Event management systems are key epidemic intelligence tools for PHI, integrating outputs of surveillance systems, combining surveillance and response workflows, and enabling oversight to streamline investigation of and response to public health threats. We describe NCDC’s SITAware, a simple, easy-to-use, and effective web-based event management system. SITAware was developed through multiorganizational cooperation and partnership, defined by user and institutional needs, and achieved with few resources and at low cost compared to de novo system developments. Although there have been challenges in embedding the system, SITAware has met a critical need at the national and state levels in Nigeria, contributing to improvements in event oversight, management, and coordination and in enabling fast and effective public health action.

We recommend other countries with existing EMS document their experiences by mapping user workflows with processes for surveillance and response, monitoring timeliness of outbreak detection and response, and ensuring the outputs meet the needs of users at multiple levels. Countries without an EMS should consider if an EMS would be beneficial. Whereas an EMS product has not been developed and scaled, we believe that Nigeria’s experiences, including drawing from our lessons learned and application of enablers for success, can inform developers and countries wishing to deploy EMS. We recommend considering the adoption of functionality contained within SITAware in the design of future EMS and using a change management approach for implementation. We have proposed a simple schema to inform approaches in similar contexts, drawing from our experience.

In conclusion, the collaboration achieved between national public health agencies and partners was essential to the creation and successful implementation of SITAware. We believe this process can serve as a model for knowledge sharing and health protection capacity building elsewhere.
